# Street protests and air pollution in Hong Kong

**DOI:** 10.1007/s10661-020-8243-0

**Published:** 2020-04-19

**Authors:** Peter Brimblecombe

**Affiliations:** 1 School of Energy and Environment, City University of Hong Kong, Kowloon Tong, Hong Kong; 2 Guy Carpenter Climate Change Centre, City University of Hong Kong, Kowloon Tong, Hong Kong

**Keywords:** Street blockage, Traffic diversion, Roadside air pollution, NO_2_, Traffic associated air pollutants (TRAP)

## Abstract

June 2019 saw large-scale street protests in Hong Kong that impeded traffic flow along streets in areas around to the Legislative Council building. These had the potential to reduce overall air pollutant emissions from traffic and lower their concentrations. Two roadside monitoring stations relatively close to the Legislative Council reveal that measured concentrations of nitrogen dioxide declined during the protests compared with measurements from other sites by at least 50% on many occasions. There were only subtle changes in particulate loads and no evidence of any reduction in carbon monoxide concentrations. Pedestrianisation and bus route rationalisation are often seen as methods to reduce exposure in congested areas, but the observations here suggest that the substantial improvements in the nitrogen dioxide levels might not be matched by improvements in other pollutants. Plans for changes to street layouts to improve air quality need careful investigation before they are implemented.

Disrupted traffic flows during large-scale street protests seem likely to affect local air quality. Although Hong Kong residents are often seen as compliant, there have been several large-scale public protests since 1997, when it became a special administrative region of China. Such protests are mainly driven by concerns over the gradual erosion of autonomy granted to the Hong Kong Special Administrative Region (HKSAR) as part of a historic agreement between China and the UK. More than seven million people live in the region, which has relatively little habitable land. Population density is therefore high, allowing public transport to be very efficient. This offers the potential for large crowds to gather rapidly, which in recent years has been enhanced by the prevalence of social media. It is possible for a million people to gather for an afternoon of protest.

Car ownership in Hong Kong is relatively low, but diesel buses and goods vehicles and taxis (using liquid petroleum gas) are important pollutant sources along the crowded roads in many urban areas. Three roadside air pollution monitoring stations in Hong Kong reveal high concentrations of traffic-derived pollutants that follow regular daily and weekly cycles. There are also seasonal changes to air pollutants with the higher concentrations found at the beginning and end of the year, when the wind speeds are low and mixing depths are reduced (Louie et al. 2005; Ai et al. 2016). Nitrogen dioxide is an important roadside pollutant in Hong Kong, often exceeding air quality objectives, which may be attributed to increasing traffic, higher primary emissions of NO_2_ (Tian et al. 2011) and a heavy vehicle fleet whose emissions are higher than expected (Lau et al. 2015). In 2014, protesters carrying yellow umbrellas blocked the streets for several months. Media articles suggested that the protests could make Hong Kong’s air cleaner, which led to an enduring view that the umbrella movement temporarily solved the cities air pollution problems (SCMP 2014, Kan, 2015; Cheung 2017). Naturally, the situation was rather more complex than this (Pan et al. 2017). Some pollutants (most notably nitrogen dioxide) were reduced in the early days of the protests, and there were subtle changes in the concentrations of fine particulate materials. Carbon monoxide changed little and ozone increased, because it was no longer titrated out by nitric oxide (Brimblecombe and Ning 2015). Nevertheless, correlation analysis revealed that the road blockage failed to change the daily cycle of PM_10_ and PM_2.5_ in the urban area (Lu et al. 2016). After the initial stages of the protest, when the nitrogen oxides were at a reduced level, air quality subsequently worsened. Part of this was simply seasonal change, although traffic was effectively redirected along alternate routes to avoid the protests (Brimblecombe and Ning 2015).

Traffic diversions of the kind imposed by protests are a useful indicator for those planning to pedestrianise or change the vehicle mix on streets. Hong Kong has been enthusiastic about pedestrianisation (HKIP 2014), bus route rationalisation and reducing oversupply of buses (Chai 2015; Tang and Lo 2008) as ways of improving air quality. However, little research has been done on the impact of such changes on air pollution. If cities are made free of cars, then there can be as much as 40% reduction in NO_2_ (Nieuwenhuijsen and Khreis 2016). The improved air quality from better traffic flow, green waves or one-way systems may be short-lived if traffic flow increases because of reduced congestion (Joumard et al. 1996). Restrictions can be applied to some urban areas such as imposed by traffic diversions (Walker et al. 1999; Clench-Aas et al. 2000) or through congestion charges, but the changes may be subtle (Beevers and Carslaw 2005; Atkinson et al. 2009). Much is written about the potential benefits of pedestrianisation (Soni and Soni 2016) to reduce air pollution (Kan 2015; Maliene et al. 2018; Yassin 2019), but these seem to derive more from enthusiasm than measured data, so further studies are clearly needed. Even though Shafray and Kim (2017) make a plausible claim of a 35% decrease of air pollution from the Cheonggyecheon restoration project in Korea, details of this achievement are sparse. As Nieuwenhuijsen and Khreis (2016) argue, freeing urban areas of cars ‘is likely to have direct and indirect health benefits, but the exact magnitude and potential conflicting effects are as yet unclear’.

This paper looks at the pollutant concentrations during some short-duration public protests in central areas of Hong Kong, using data available from Hong Kong’s Environmental Protection Department (HKEPD) monitoring stations. The sudden changes in pollutant levels that occur when streets are blocked during large protests give an opportunity to explore the changes that could arise if traffic reduction plans were implemented (Pan et al. 2017). This work extends a previous study (Brimblecombe and Ning 2015), which examined the lengthy protest of 2014, examining the brief demonstrations that occurred on a number of days in the early summer of 2019. Later in the year, protests were often smaller and widely spread across the region, so they were more difficult to study. However, it is useful to compare 2019 with some previous events (> 2003) in the central parts of Hong Kong. This study aims to draw lessons about the improvements to air quality and considers how these might be used to assess likely air quality improvements from pedestrianisation or altering transport flow along streets.

## Method and data sources

The public protests in Hong Kong over the period 2003–2019 mentioned here are listed in Table 1. Some started with smaller events, but public participation grew over time and ultimately led to large gatherings. The number of participants is often disputed. The lower numbers in the table refers to the official values, from the police, which try to represent the maximum number of people at the peak of the gathering. The larger values are estimates from the organisers and aim to account for the total number of people involved and appear to be confirmed by independent observers.

**Table 1:** Dates and size of protests

Date	Protest form	Participation	Location
2003 National Security (Legislative Provisions) Bill 2003 protests			
1 July^a^ 14:30–22:00	Annual march	350–700k	Victoria Park to Tamar
2012 Patriotic education curriculum protests			
29 July^b^	March	36–100k	Victoria Park to Tamar
8 September^c^	Gathering	36–120k	Government HQ inTamar
2014 umbrella revolution			
26 Sept–15 Dec^d^	Street sit-in	> 100k	Central, Causeway Bay and Mong Kok
2019 Hong Kong anti-extradition bill protests^e^			
31 March	March	5–12k	Luard Road to Civic Square
28 April	March	23–130k	Causeway Bay to Legislative Council
9 June	March	0.23–1.03M	Victoria Park to Tamar
12 June	Sit-ins		Government HQ, Tamar
16 June	March	0.34–1.44M	Victoria Park to Tamar
21 June	Street rally		Government HQ, Tamar; then police HQ
26/27 June^f^	Street rally		City Hall and police HQ
30 June^g^	Gathering	165k	Tamar Park
1 July^h^	March, occupation	190–500k	Victoria Park to Tamar; Legislative building

References

(a) https://en.wikipedia.org/wiki/Hong_Kong_1_July_marches#2003 —March scheduled for 2:30 pm in Victoria Park, but people were still starting the march as late as 10 pm.

(b) https://news.yahoo.com/thousands-hk-protest-china-patriotism-classes-102106600.html

(c) http://world.time.com/2012/09/10/why-hong-kong-wants-nothing-to-do-with-patriotism-for-now

(d) Ng (2016), Brimblecombe and Ning (2015)

(e) https://en.wikipedia.org/wiki/2019_Hong_Kong_anti-extradition_bill_protests

(f) https://time.com/5614357/hong-kong-g20-extradition-protest-democracy-foreign-intervention/

(g) https://www.bbc.com/news/world-asia-48817530 Pro-Beijing opposition to the anti-extradition protests and support for the police Hong Kong.

(h) https://en.wikipedia.org/wiki/2019_Hong_Kong_anti-extradition_bill_protests#Protests_of_1_July

**Fig. 1:**
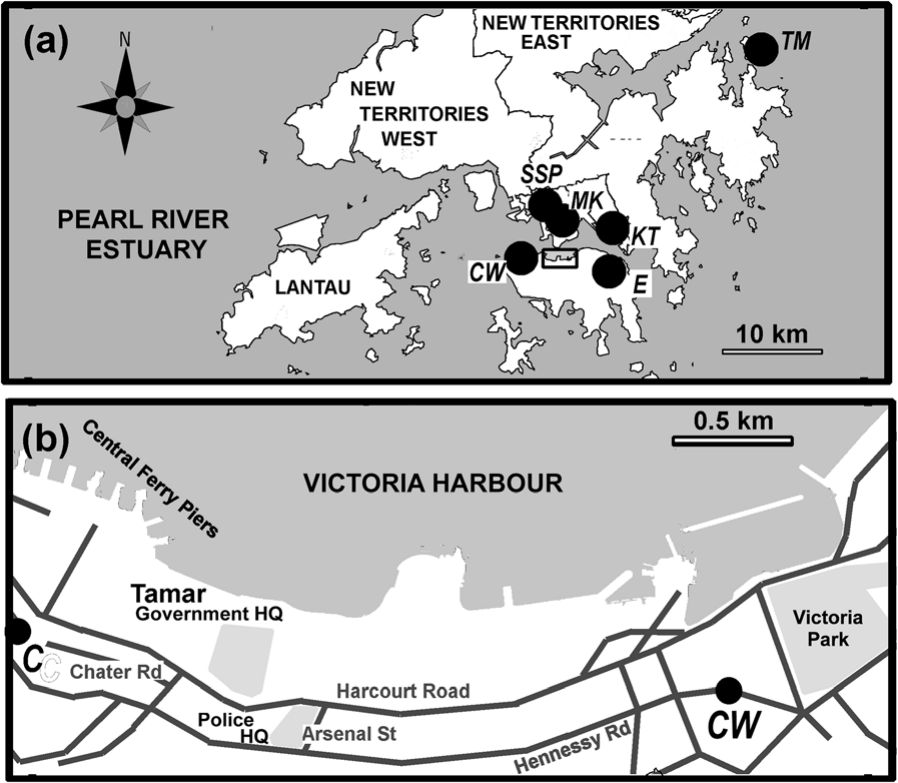
Map of Hong Kong showing the study area. **a** Shows much of Hong Kong, its New Territories and the outer islands along with HKEPD monitoring stations at Sham Shui Po (SSP), Mong Kok (MK) a roadside station, Kwun Tong (KT), Central and Western (CW) and Eastern (E) which are marked as black dots. The black rectangle is expanded as Fig. 1b with the two roadside sites on Hong Kong Island, Causeway Bay (CB) and Central (C). Major roads are marked in dark grey along with important sites; Victoria Park where marchers usually gather and move along Hennessey Road to various official headquarter buildings, that at the Central Government Complex at Tamar adjacent to Harcourt Road and the Hong Kong Police Headquarters on Arsenal Street

The location of the protests and the nearby HKEPD monitoring sites are shown in Fig. 1. Data from these stations are available at http://epic.epd.gov.hk/EPICDI/air/station/?lang=en. General stations, effectively urban background sites, are present at (i) Central and Western (CW), and (ii) Eastern on Hong Kong Island, and (iii) Kwun Tong (KT) and (iv) Sham Shui Po (SSP) in Kowloon East and West (see Fig. 1a). Additionally, there is a background site on Tap Mun (TM), an isolated island off the east coast of the Hong Kong Special Administrative Region. Protests typically take place on Hong Kong Island and often move along a route from Victoria Park in Causeway Bay to the Central Government Complex at Tamar or other key buildings in Admiralty, which is the eastern extension of the central business district. There are roadside monitoring sites at Causeway Bay (CB) and Central (C), as marked on the map in Fig. 1b. These are effectively the eastern and western extremes of potential protest disruption to traffic. A further roadside monitoring site is found at Mong Kok (MK) on the Kowloon Peninsula across the Victoria Harbour (Fig. 1a).

The HKEPD monitoring sites provide hourly measurements of NO_2_, NO_x_, CO, O_3_, PM_2.5_ and PM_10_, although CO measurements are not available for the four general stations. It was necessary to make comparisons between the measurements from other sites and those close to the protests at Causeway Bay and Central to assess likely concentrations of pollutants in the absence of the protests. Pollutant concentrations between sites in Hong Kong are often reasonably well-correlated, so that concentrations at Causeway Bay and Central can be determined through multiple regression with neighbouring sites, using least squares fits to an equation of the form:

1 $$ {c}_P={a}_0+{a}_{MK}{c}_{MK}+{a}_{CW}{c}_{CW}+{a}_E{c}_E+{a}_{KT}{c}_{KT}+{a}_{SSP}{c}_{SSP}\kern2.25em \dots $$

where *c*_P_ represents the protest sites of Causeway Bay and Central, *a*_0_ the intercept and *a*_MK_ the regression parameter, and *c*_MK_ the concentration for Mong Kok and additionally the other nearby sites. During protests, the multiple regression equation can be used to estimate likely concentrations were there are no protests. A similar approach was successfully applied to monitoring data in earlier work from these sites (Brimblecombe and Ning 2015) and relies on the observation that nearby monitoring sites tend to be well-correlated. Although such regression equations are quick to apply, they do not allow us to understand the importance of day-to-day variation in wind speed, temperature and atmosphere stability on the reduced concentrations observed. The multiple regression used the online tool Wessa.net (Wessa, 2020), and was applied to the data set over months that bracketed the protests. The chi squared test was used to assess the differences in pollutant concentrations between the protest and non-protest periods using an online software (https://www.socscistatistics.com/tests/chisquare/default2.aspx).

## Results

### NO_2_ concentrations during the anti-extradition protests 2019

The 2019 Hong Kong anti-extradition bill (formally the Fugitive Offenders and Mutual Legal Assistance in Criminal Matters Legislation (Amendment) Bill) protests in June 2019 were short-lived events so differed from the umbrella movement occupation of 2014. The latter took place over 79 days from late September, when pro-democracy advocates were present in three locations across Hong Kong (Ng, 2016). The shorter-length street protests of 2019 allow us to explore the effect of rapid changes in traffic flow. Bus companies were aware of the likely impact on traffic flow, so they planned route diversions and bus stop relocations for June 9, June 16 and July 1 (e.g. http://www.kmb.hk/en/news/press/archives/news201906062792.html).

**Fig. 2:**
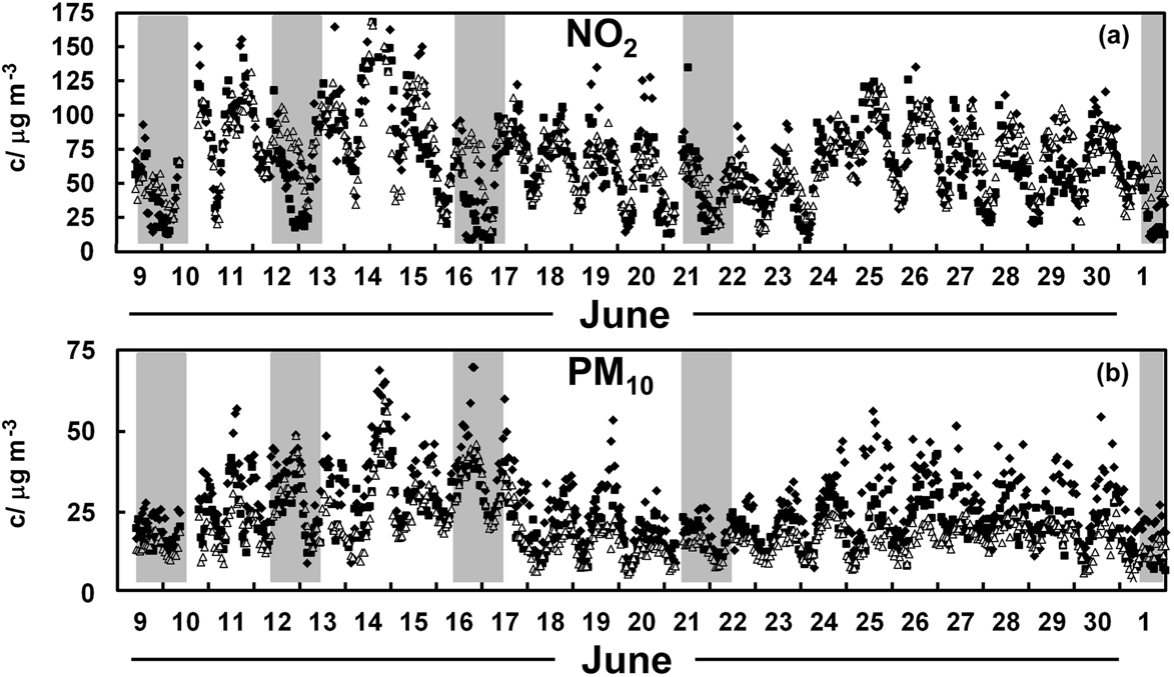
Measured NO_x_ concentrations (c) at the roadside sites in Causeway Bay (filled diamonds), Central (filled squares) and Mong Kok (open triangles) covering the protests of June 2019. The shaded areas mark afternoon and evenings of protests

Figure 2 a displays the nitrogen dioxide concentration over June and illustrates that the NO_2_ concentrations in Causeway Bay and Central were typically depressed when compared with the unaffected roadside site in Mong Kok over key periods of protest, as marked with grey shading. There is little obvious improvement to PM_10_ concentrations during the protest periods across the month of June (Fig. 2b). Any reductions that seem apparent in Fig. 2 are not easily attributed to the street blockages as the low pollutant levels might be the result of meteorological factors. This problem was addressed by estimating the likely concentrations of pollutants in Causeway Bay and Central based on concentrations found in other monitoring stations as successfully applied in an earlier work (Brimblecombe and Ning 2015). Parameters established from multiple linear regression of data for the months around the protest are used to predict expected concentrations during the protests. These estimates can be compared with the measured values and allow us to assess whether the demonstrations that blocked traffic lowered pollutant concentrations, such that measured air pollutant concentrations were lower than the estimated values.

**Fig. 3:**
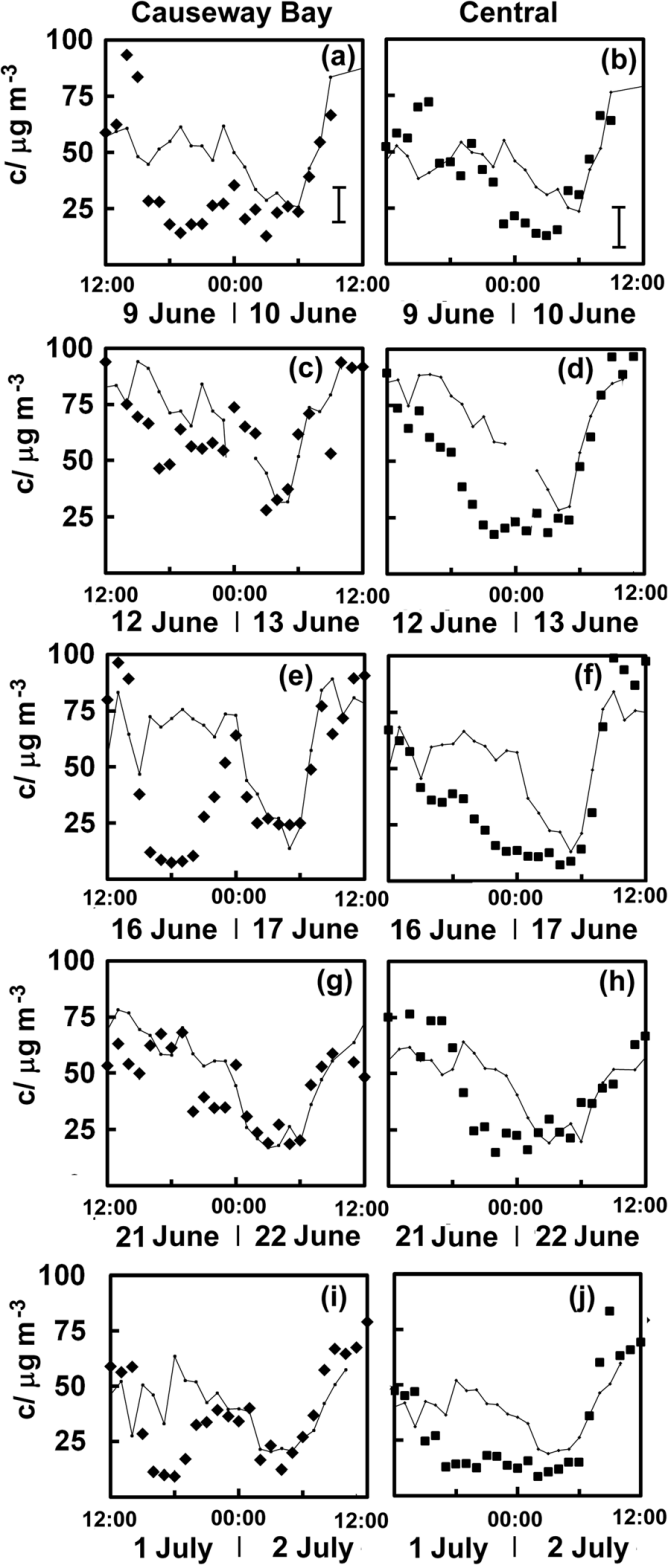
Measured NO_2_ concentrations (c) and estimated values (lines joining small squares for Causeway Bay (filled diamonds) and Central (filled squares) for 2019 protests on the 9th of June (**a**, **b**), 12th of June (**c**, **d**), 16th of June (**e**, **f**), 21st of June (**g**, **h**) and 1st of July (**i**, **j**). Note that estimated values come from the equations *C*_CB_ = 0.45 + 0.86*c*_MK_ − 0.11*c*_CW_ + 0.48*c*_E_ + 0.27*c*_KT_ − 0.38*c*_SSP_, with *R*^2^ = 0.85 and *C*_C_ = 2.39 + 0.42*c*_MK_ + 0.06*c*_CW_ + 0.46*c*_E_ + 0.3*c*_KT_ + 0.14*c*_SSP_, with *R*^2^ = 0.83, where *C*_CB_ and *C*_C_ are the predicted concentrations at the two measurement sites near the protests and c is the concentration measured at other sites on Hong Kong and the Kowloon Peninsular. The standard deviation of the residuals from the fitting equations is marked as error bars in the lower right of Fig. 3 a and b

Figure 3a shows the NO_2_ concentrations measured at the Causeway Bay (CB) station on 9 June 2019 as black diamonds, while the fine line joining small back squares represents the predicted values as estimated from Eq. (1), using the fitting parameters and *R*^2^ noted in the caption to Fig. 3. The standard deviation of the residuals from the fitting equation is marked as an error bar in Fig. 3a. It is evident that the concentrations were much lower than expected from early afternoon to almost midnight. Protesters, concerned with the potential issues raised by the extradition bill, had gathered in Victoria Park around 13:00 on the 9th of June. This was a little before the march was to begin at 15:00, perhaps in part due to fears that public transport would be very crowded. There proved to be so many people that it was hard for the demonstrators to move forward, and many were much delayed in exiting the park. Thus, it is not surprising that NO_2_ concentrations remained lower than expected until nearly midnight. Around a million people were likely to have made their way towards Tamar, and crowds began to congest the streets nearby reducing traffic emissions. By the evening, lower than expected levels of NO_2_ were found at the roadside monitoring site in Central (C), which continued until the early hours of the morning (Fig. 3b). Even though the march formally ended at 22:00, hundreds of protesters camped out in front of the government headquarters well into the night, with more joining midst flying bottles and pepper spray.

Although the Government acknowledged that there were concerns over the extradition bill, it refused to withdraw the legislative plans. Trade unions, religious groups and students gathered on June 12 to protest about the second reading of the bill. Anti-riot police began to gather around 13:00, and shortly after, the demonstration was declared a riot by the Commissioner of Police. This justified the use of tear gas, pepper spray, rounds of rubber bullets and beanbag shots to break up the crowds, but protesters dispersed slowly. The monitoring record and estimates suggest no evidence of any decrease in NO_2_ to the east at Causeway Bay (Fig. 3c). This location played no role in the demonstrations, which took place in Tamar, so affected air pollution at the roadside site in Central (Fig. 3d), where we can see lower than expected NO_2_ concentrations late into the night.

Sunday, June 16 saw another massive protest, even larger than that of the week before. More than a million people marched from Victoria Park to Tamar. There were unexpectedly low concentrations of NO_2_ at Causeway Bay through the latter half of the day (Fig. 3e) and subsequently lower concentrations at the Central site (Fig. 3f) as the crowd occupied all the six lanes of Hennessy Road and moved onto parallel streets. Protestors remained until early the following morning as reflected in the persistence of unexpectedly low concentrations.

Another protest took place on June 21, with demonstrators gathering outside the government headquarters demanding the complete withdrawal of the extradition bill, blocking traffic on Harcourt Road soon after 11:00. There had also been complaints about overreaction from the police, so some of the protesters marched to the Hong Kong Police Headquarters remaining there until well into the evening. There is no strong evidence of any lower than expected NO_2_ concentrations at the Causeway Bay site (Fig. 3g) as no substantial gatherings had formed at Victoria Park. However, lowered NO_2_ concentrations, which persist late into the night, appear at the roadside site in Central (Fig. 3h).

July 1 is Establishment Day in Hong Kong and commemorates the transfer of sovereignty from the UK to China. It has become a day of protest rallies in the twenty-first century and though these are predominately in support of democracy, although there are often demonstrations by pro-Beijing groups. As listed in Table 1, both groups were represented in 2019, although on June 30, the pro-Beijing group supporting the police simply gathered in the park in Tamar. The annual democracy march on July 1 was largely peaceful, but some protesters began to storm the Legislative Council building later in the day, so the main march diverted to Chater Road. As with the earlier marches of June 9 and 16, concentrations of NO_2_ were lower than expected at the Causeway Bay site (Fig. 3i) in the period when the marchers assembled in Victoria Park. Lowered values persisted longer at the Central monitoring site (Fig. 3j).

### Particulate matter and carbon monoxide, 2019

**Fig. 4:**
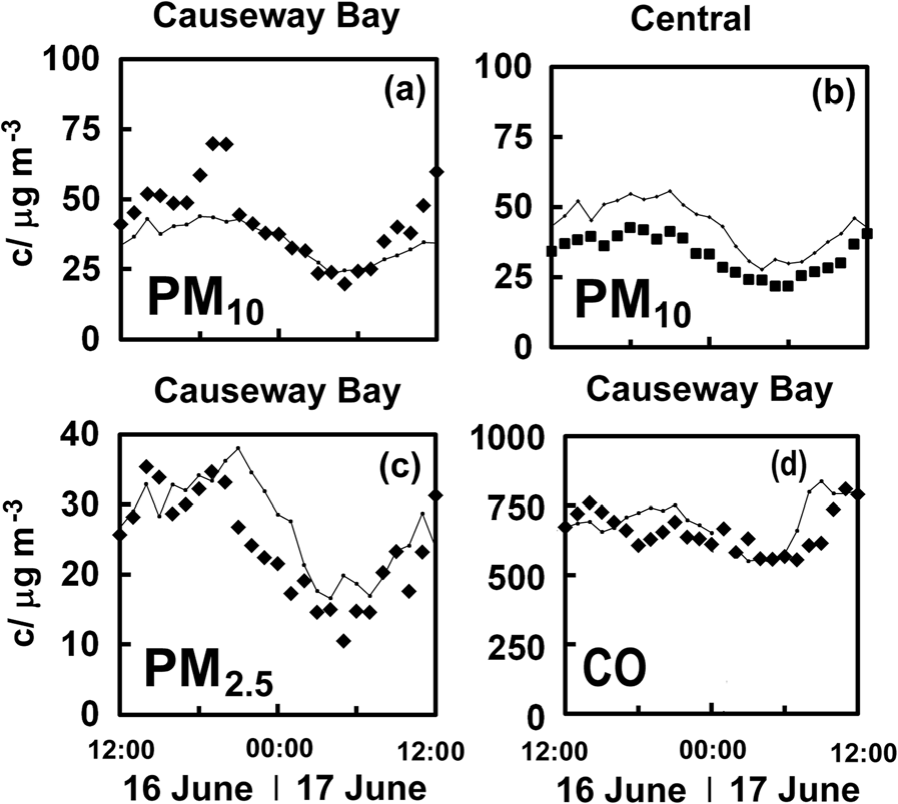
Measured PM_10_ concentrations (c) and estimated values (lines joining small squares) for (**a**) Causeway Bay (filled diamonds) and (**b**) Central (filled squares) for protests of 16 June 2019. PM_10_ (**c**) and carbon monoxide (**d**) concentrations and estimated at Causeway Bay. Note that estimated values come from the equations (**a**) *C*_CB,PM10_ = 5.83 + 0.35*c*_MK_ + 0.38*c*_CW_ − 0.08*c*_E_ + 0.15*c*_KT_ + 0.11*c*_SSP_, *R*^2^ = 0.78; (**b**) *C*_C,PM10_ = 7.33 + *c*_MK_ + 0.10*c*_CW_ − 0.28*c*_E_ + 0.07*c*_KT_ + 0.18*c*_SSP_, *R*^2^ = 0.67; (**c**) *C*_C,PM2.5_ = 5 + 1.02*c*_MK_ − 0.06*c*_CW_ − 0.28*c*_E_ + 0.11*c*_KT_ + 0.12*c*_SSP_, *R*^2^ = 0.78; (d) *C*_C,CO_ = 237 + 0.4*c*_MK_ + 0.11*c*_TM_ + 1.26*c*′_CW_ + 3.46*c′*_E_ + 1.84*c*′_KT_, *R*^2^ = 0.85 where primes denote using NO_2_ concentrations rather than CO at sites where it is not measured.

Other pollutants show less evident changes as suggested for PM_10_ in Fig. 2b. The most obvious change in NO_2_ concentrations occurred during the events of 16 June 2019, especially at Causeway Bay (Fig. 3e), so this seems the appropriate location to search for effects on other pollutants. Figure 4 shows PM_10_, PM_2.5_ and CO at roadside sites, but the evidence for an effect from the protests is much less convincing. The levels of PM_10_ at the sites in Causeway Bay and Central (Fig. 4a–b) show little evidence of lowered concentrations over the time when NO_2_ concentrations there proved much lower than expected. It was anticipated that a noticeable effect would emerge for PM_10_ which is more heterogeneously distributed in Hong Kong than PM_2.5_, but it was not obvious. There may be a hint of lower than expected levels of PM_2.5_ (Fig. 4c), but the timing of this is rather later than that of the decrease in NO_2_ at Causeway Bay. The estimated levels of CO are more of a problem to estimate because its concentrations are not measured at the four general stations, so some NO_2_ values were used in the regression equation along with CO from Mong Kok and Tap Mun (see caption of Fig. 4d for details). This figure fails to provide convincing support for lowered levels of CO. This difficulty in finding a clear signal for the effect of protests on pollutants other than the nitrogen oxides is much in line with observations made during the umbrella movement of 2014 (Brimblecombe and Ning 2015).

### Annual 1st of July protest 2003

**Fig. 5:**
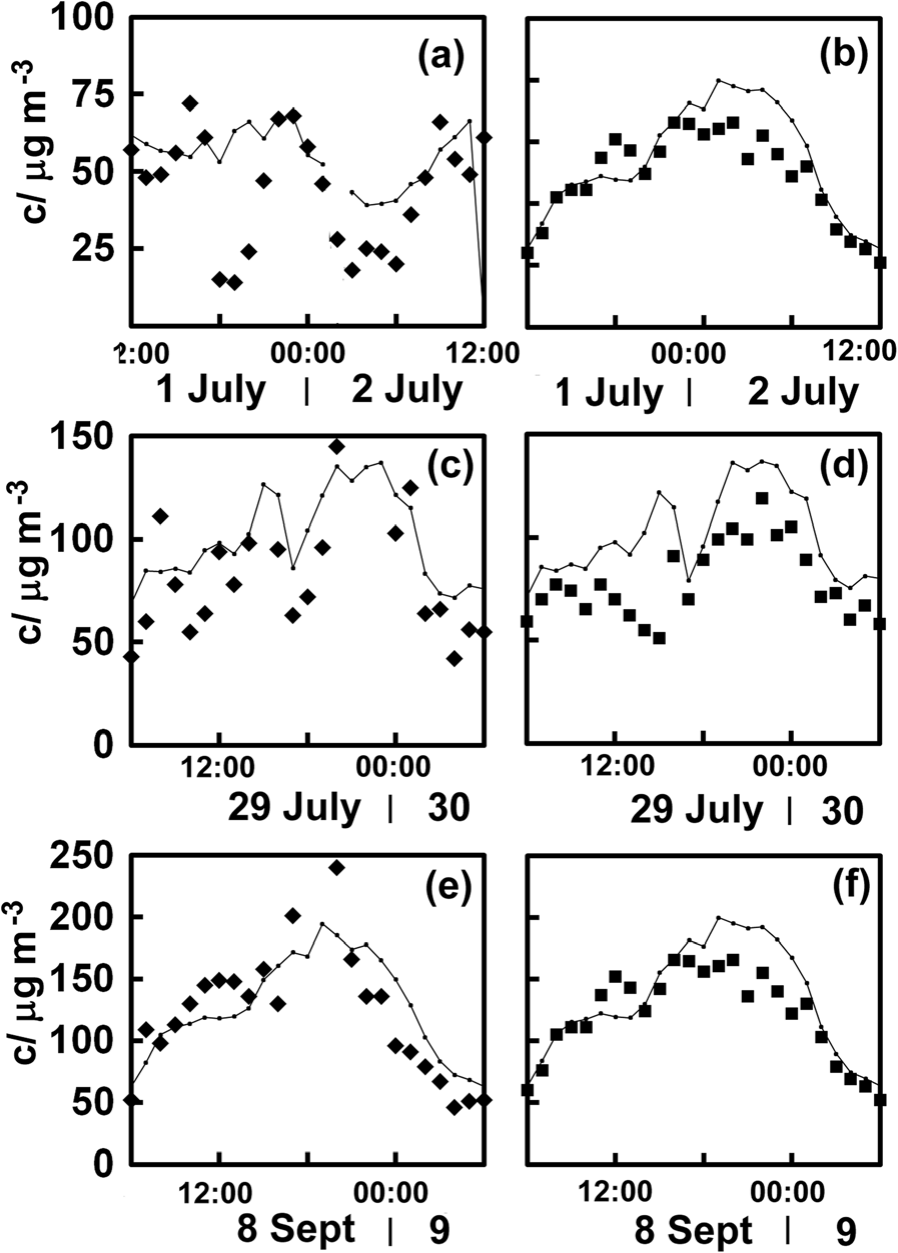
Measured NO_2_ concentrations (c) and estimated values (lines joining small squares for Causeway Bay (filled diamonds) and Central (filled squares) for protests of 1 July 2003(**a**, **b**), 29 July 2012 (**c**, **d**) and 8 September 2012 (**e**, **f**). Note that estimated values come from the equations (**a**) *C*_CB_ = 6.9+ 0.38*c*_MK_ − 0.05*c*_CW_ + 0.6*c*_E_ + 0.24*c*_KT_ − 0.05*c*_SSP_, *R*^2^ = 0.79; (**b**) *C*_C_ = 11 + 0.43*c*_MK_ + 0.18*c*_CW_ + 0.28*c*_E_ + 0.42*c*_KT_ − 0.13*c*_SSP_, *R*^2^ = 0.78; (**c**, **e**) *C*_CB_ = 10.5 + 0.48*c*_MK_ + 0.47*c*_CW_ + 0.27*c*_E_ + 0.27*c*_KT_ − 0.11*c*_SSP_, *R*^2^ = 0.68; (**d**, **f**) *C*_C_ = 3 + 0.39*c*_MK_ + 0.53*c*_CW_ + 0.37*c*_E_ + 0.21*c*_KT_ + 0.14*c*_SSP_, *R*^2^ = 0.77

It seemed likely that earlier protests would also have led to a similar depression of the NO_2_ concentrations. The annual gathering on July1, Hong Kong’s Establishment Day, was especially large in 2003 because of concerns over the 2003 National Security (Legislative Provisions) Bill. As many as 0.7 million people are believed to have marched from Victoria Park to Tamar. We can see a sharp decrease in observed concentrations compared with those expected in the late afternoon at Causeway Bay (Fig. 5a) and a small decrease in concentrations over those expected somewhat later in the measurements from Central (Fig. 5b), although it is not very convincing.

### Patriotic education curriculum protests 2012

Concern over changes to the school curriculum in Hong Kong caused a series of popular demonstrations in 2012 from those who feared the curriculum would be used to encourage children to support the Chinese Communist Party. The march of July 29 from Victoria Park to Tamar involved as many as a hundred thousand people. There is little evidence of depressed NO_2_ concentrations at the site in Causeway Bay (Fig. 5c), but at the monitoring site in Central, concentrations were lower throughout the afternoon (Fig. 5d). Pressure on the government to drop these plans continued, with the new government headquarters at Tamar surrounded by dozen of activists who began hunger strikes in tents through the first days of September. Matters reached ahead September 8, when around 100,000 people gathered chanting slogans and listening to speeches that denounced the national education plans. Understandably, the NO_2_ concentrations at the distant Causeway Bay station (Fig. 5e) seemed unaffected, but the concentrations were lowered somewhat at Central, until well into the night.

## Discussion and conclusion

The data above suggests that when the streets were blocked by protestors, the NO_2_ concentrations were lower than expected. However, it is fair to ask whether lower than expected concentrations could occur randomly at times when there are no street blockages. The concentrations at the roadside monitoring site in Causeway Bay and Central for June 9 to July 1 were examined for cases where the measured concentrations were less than half those estimated in both the protest periods (i.e. 12:00 to 06:00 the following day) and the periods where there were no nearby protests. At Causeway Bay, 12 h met this criterion, while 48 failed. During the periods without protests, 17 h met the criterion, while 445 failed; at Central, the number of hours was 28, 32, 11 and 511 respectively. A chi squared analysis using a 2 × 2 contingency determined that for both sites, the population of measurements was not independent at the 99% confidence level. This suggests that the observations made during protest periods are drawn from different populations from that when there were no protests.

During the June 2019 protests, NO_2_ concentrations appeared to be reduced by some 50% for 4–6 h, with these periods representing a reduction of about 36 μg m^−3^. This can be compared with smaller reductions in excess of 36 μg m^−3^ at Causeway Bay in 2003 and similarly modest decreases in NO_2_ at Central on 29 July 2012. In 2014, the protest site in Central saw major reductions (~ 50 μg m^−3^ representing about a 50% decrease) the early days of the street blockages, but only subtle changes in PM_10_ (Brimblecombe and Ning 2015). The long-term occupations of 2014 allowed a gradual reassertion of traffic flow, so the pollutant reductions gradually declined as traffic adopted other nearby routes. That was less likely for the shorter protests as seen in the early summer of 2019.

Ai et al. (2016) suggest that along busy roads in Hong Kong, NO_2_ may meet the 1-h standards, while particulate matter is likely to exceed the safe limits. Observations during the protests suggest the most obvious reductions are those for NO_2_ and less so for the particulate matter, which is a key driver of urban health. Although changes in traffic flow down a street may decrease NO_x_ concentrations, this may be less effective with air pollution in general, which is likely to be more homogeneously spread, unless rather extensive areas are made vehicle free. Efforts to model the impact of pedestrianisation have suggested significant (70–80%) reductions in CO, hydrocarbons, NO_x_, PM and CO_2_ (Chiquetto 1997) making the measure appear much more favourable than suggested by the measurements presented here. Regardless of this it is important to look carefully at the likely effects of pedestrianisation and traffic diversion, perhaps through modelling, before embarking on extensive changes to traffic routing.
